# *Alpinia* Essential Oils and Their Major Components against* Rhodnius nasutus*, a Vector of Chagas Disease

**DOI:** 10.1155/2018/2393858

**Published:** 2018-02-15

**Authors:** Thamiris de A. de Souza, Marcio B. P. Lopes, Aline de S. Ramos, José Luiz P. Ferreira, Jefferson Rocha de A. Silva, Margareth M. C. Queiroz, Kátia G. de Lima Araújo, Ana Claudia F. Amaral

**Affiliations:** ^1^Faculty of Pharmacy, Federal Fluminense University, Rua Doutor Mário Viana 523, Santa Rosa, Niterói, RJ, Brazil; ^2^Laboratory of Medicinal Plants and Derivatives, Department of Chemistry of Natural Products, Farmanguinhos, FIOCRUZ, Rio de Janeiro, RJ, Brazil; ^3^Laboratory of Medical and Forensic Entomology, IOC, FIOCRUZ, Av. Brasil 4365, Rio de Janeiro, RJ, Brazil; ^4^Chromatography Laboratory, Chemistry Department, Federal University of Amazonas, Manaus, AM, Brazil

## Abstract

Species of the genus* Alpinia* are widely used by the population and have many described biological activities, including activity against insects. In this paper, we describe the bioactivity of the essential oil of two species of* Alpinia* genus,* A. zerumbet* and* A. vittata*, against* Rhodnius nasutus*, a vector of Chagas disease. The essential oils of these two species were obtained by hydrodistillation and analyzed by GC-MS. The main constituent of* A. zerumbet* essential oil (OLALPZER) was terpinen-4-ol, which represented 19.7% of the total components identified. In the essential oil of* A. vittata* (OLALPVIT) the monoterpene *β*-pinene (35.3%) was the main constituent. The essential oils and their main constituents were topically applied on* R. nasutus* fifth-instar nymphs. In the first 10 min of application, OLALPVIT and OLALPZER at 125 *μ*g/mL provoked 73.3% and 83.3% of mortality, respectively. Terpinen-4-ol at 25 *μ*g/mL and *β*-pinene at 44 *μ*g/mL provoked 100% of mortality. The monitoring of resistant insects showed that both essential oils exhibited antifeedant activity. These results suggest the potential use of* A. zerumbet* and* A. vittata* essential oils and their major constituents to control* R. nasutus* population.

## 1. Introduction

**Figure 1:**
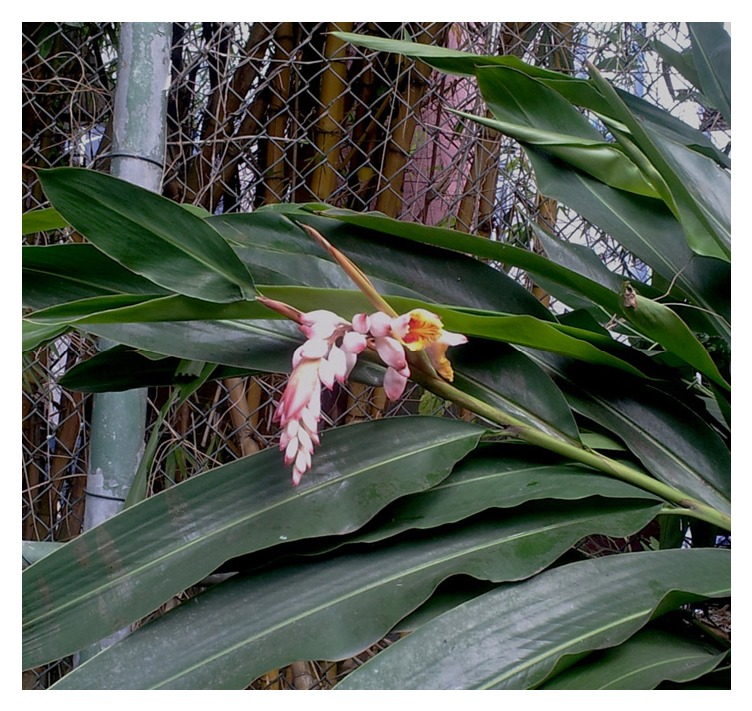
*Alpinia zerumbet* (Pers.) Burtt & Smith.

**Figure 2:**
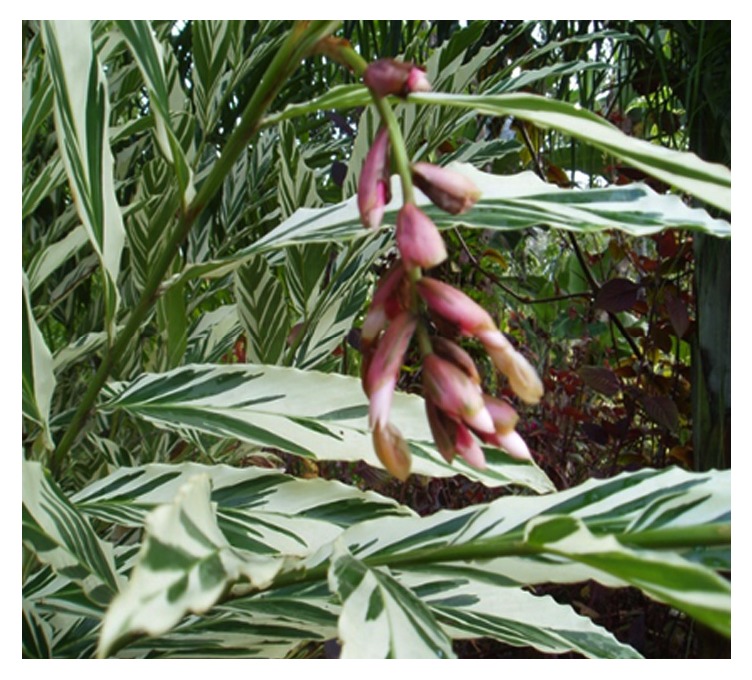
*Alpinia vittata* W. Bull.

Preparations from the leaves of* Alpinia zerumbet* (Pers.) Burtt & Smith (family Zingiberaceae), also known as* Alpinia speciosa* (J. C. Wendl.) K. Schum. or* Alpinia nutans* (L.) Roscoe [1] (Figure 1), are used as sedative and for reducing blood pressure [2]. Another species of the same genus,* A. vittata* W. Bull (Figure 2), presents different physical characteristics, although some chemical constituents and biological activities are similar. Polyphenols such as kaempferol, quercetin, myricetin, isorhamnetin, flavone C-glycosides, and proanthocyanidins have been isolated from* Alpinia* species [3]. These constituents are antiallergic, antiatherogenic and anti-inflammatory, antimicrobial, antithrombotic, cardioprotective, and vasodilating agents [4]. They are also useful in the food and pharmaceutical industry because they can be used as substitutes for potentially carcinogenic synthetic antioxidants [5].

Essential oils from species of* Alpinia* genus have activity against insects, which makes them prototypes for formulations with insecticidal action [6–10]. In this context, a solution of 0.1% of the essential oil from* A. zerumbet* flowers exhibited repellent activity (51.8%), irritating action (64.9%), and an interesting toxic activity (66.8%) against the mosquito* Aedes aegypti*, suggesting that this oil could be evaluated as bioinsecticide against this vector. The main components of the* A. zerumbet* flowers oil, 1,8-cineol and terpinen-4-ol, exhibited repellent action against* A. aegypti* [11], whereas p-cymene and *α*- and *γ*-terpinene displayed strong larvicidal properties [11–13]. Additionally, the monoterpene sabinene displays to some extent biting deterrent activity against this mosquito species [6, 13].

Also regarding the insecticidal activity, some of the main components found in the essential oil of* A. zerumbet* and other plant species, notably 1,8-cineol and terpinen-4-ol, exhibited activity against hematophagous insects such as* Triatoma infestans *and* Rhodnius prolixus*, belonging to the family Reduviidae [14–18]. These insects are vectors of the hemoflagellate protozoa* Trypanosoma cruzi* and responsible for the transmission of Chagas disease, also known as American trypanosomiasis. This disease affects around 6 million people worldwide, especially in Latin America, and causes approximately 14,000 deaths annually. So far, there is no preventive vaccine or a suitable treatment in its chronic phase [19].

Triatomines are necessarily hematophagous and belong to the genera* Triatoma*,* Panstrongylus,* and* Rhodnius* [20]. According to Lima et al. [21],* Rhodnius* genus has several species that are important vectors for* T. cruzi*, such as* Rhodnius nasutus* Stal. This species has been found in human habitations and its main control uses synthetic insecticides. However, residues generated by insecticides can cause harm to the environment, humans, and animals [22]. Therefore, in view of the development of bioinsecticides, this work aimed to study the activity of the essential oils and their major constituents of two species of* Alpinia* genus on* Rhodnius nasutus*, an important vector of Chagas disease.

## 2. Material and Methods

### 2.1. Plant Material

Leaves of* A. zerumbet* and* A. vittata* were collected in a cultivated area of Oswaldo Cruz Foundation (FIOCRUZ) in Rio de Janeiro/RJ, Brazil, on May 2016. Botanical identification was performed by Dr. Marcelo Neto Galvão of Farmanguinhos/FIOCRUZ. The vouchers were deposited at the Botanical Collection of Farmanguinhos/FIOCRUZ, with the identification numbers CBPM 672 and 675, respectively. Cut fresh leaves were measured using a pachymeter. Fragments measured about 78.9 ± 22.6 mm^2^.

### 2.2. Essential Oils

The essential oils from fresh leaves of* A. zerumbet* (OLALPZER) and* A. vittata* (OLALPVIT) were obtained by hydrodistillation using a modified Clevenger apparatus for 2 h.

### 2.3. Gas Chromatography Coupled to Mass Spectrometry (GC-MS) Analyses

Samples were analyzed by GC-MS on an Agilent 6890N GC coupled to a quadripolar mass spectrometer (Agilent 5973N) with ionization by electronic impact (70 eV). The apparatus was fitted with a DB-5MS column (30 m × 0.25 mm I.D., 0.25 *μ*m phase film). Injected volume was 1 *μ*L, in splitless mode. The injector temperature was 250°C, ion-source was 230°C, and the scan-range was 40–700 Daltons. The oven temperature varied from 40°C to 300°C, at a rate of 4°C/min. Helium was the carrier gas with a flow rate of 0.5 mL/min. Interpretation and identification of fragmentation mass spectra were carried out by comparison with the Wiley NBS mass spectrum data base. The results were expressed as relative percentage of peak area in chromatogram.

### 2.4. Biological Assays against* R. nasutus*

The oils OLALPZER and OLALPVIT diluted in DMSO were topically applied by contact to abdomen (ventral portion) of* R. nasutus* fifth-instar nymphs (1 *μ*L/insect). Insects were distributed in four groups: treated with OLALPZER, treated with OLALPVIT, without treatment, and DMSO control. After the determination of LC_50_ and LC_90_, the concentration of 125 *μ*g/*μ*L was selected for assays with groups of 30 insects, subdivided in three groups of 10 insects. The insects were kept in test tubes covered with nylon with temperature ranging from 24 to 30°C and air humidity around 60–85%. A piece of filter paper was inserted into each tube to support the locomotion of the insects. The groups of insects were weighed in analytical balance before and after the treatments. In addition, insects were also weighed before and after feeding using albino mice once a week. During the first week of study, insects were observed after 24, 48, and 72 h after application of samples. In the following weeks, insects were observed every two days. The mean weight loss, longevity, mortality, duration, and viability of the fifth instar were observed. The stages were kept under laboratory conditions with temperature ranging from 24 to 30°C and air humidity around 60–85%. Throughout the period, the mean weight loss, longevity, duration, and viability of the fifth instar and mortality of these insects were observed. Insects without motor activity or with the aid of stimuli-response were considered dead [23]. In these same conditions, the main constituents of these essential oils, 1,8-cineol, terpinen-4-ol, *α*-pinene, and *β*-pinene, purchased from Sigma-Aldrich, were tested for* R. nasutus* mortality evaluation at the same concentration that they were present in the essential oils.

### 2.5. Statistical Analysis

The results were analyzed by the Cluster analysis for index similarity according to Paleontological Statistics (PAST). Means were compared by the Tuckey-Kramer test (*p* ≤ 0.05). The software POLO was used for the determination of LC_50_ and LC_90_ by Probit analysis.

## 3. Results and Discussion

The GC-MS analyses of the essential oils extracted from fresh leaves of* A. zerumbet* (OLALPZER) and* A. vittata* (OLALPVIT) are shown in Table 1. Terpinen-4-ol and 1,8-cineol were the main compounds of OLALPZER, representing 19.7% and 15.3% of the essential oil, respectively. *β*-Pinene and *α*-pinene were the main constituents of OLALPVIT, representing 35.3% and 10.1%, respectively.

After the chemical characterization, the biological activity of the essential oils was preliminarily assayed against groups containing five* R. nasutus* fifth-instar nymphs at the concentration of 62.5, 125, and 250 *μ*g diluted in 1 *μ*L DMSO. The insects treated with OLALPZER and OLALPVIT at 250 *μ*g/*μ*L died immediately, while the insects treated with the same oils at 62.5 *μ*g/*μ*L remained alive for six weeks. OLALPZER exhibited LC_50_ and LC_90_ of 71.9 and 139.6 *μ*g/*μ*L, respectively. The LC_50_ and LC_90_ of OLALPVIT were 78.8 and 171.9 *μ*g/*μ*L, respectively. Thus, the concentration of 125 *μ*g/*μ*L for both oils was selected for the study with groups of 30 insects.

The resistant insects of the groups treated with essential oils at 125 *μ*g/*μ*L had their feeding process affected, resulting in a possible antifeedant effect of these oils, based on other studies described in the literature [24]. Control without treatment and DMSO control groups presented the total blood intake of 2988 mg and 1847 mg, respectively. Significant difference was not observed between the control groups. However, the insects of the groups treated with OLALPVIT and OLALPZER did not feed during the six weeks of observation (Table 2), representing a significant difference when compared to controls.

Some works describe the weight loss in the nymphal stage of* Triatoma pseudomaculata* [25] and* R. nasutus* [22] as more evident in the first 24 h after feeding. As expected, in the present study, the weight loss of the insects was also greater after the first 24 hours. This demonstrates that the survival capacity of triatomines for long periods without food can be a defense mechanism against insecticides, and thus these insects protected themselves until the lethal doses [26]. It is possible to suggest that the chances of a recolonization are increased by resistant insects and thus favoring the disease cycle [27].

Regarding the time necessary for fifth-instar nymphs to reach the adult stage, controls and the group treated with OLALPZER did not show important differences when considering the resistant insects (Table 3). The differences on longevity of* R. nasutus *adults were also not significant among control without treatment, DMSO control, and the group treated with OLALPZER. The insects treated with OLALPVIT died before reaching adult stage, making it impossible to calculate the time for development and longevity of this group.

**Figure 3:**
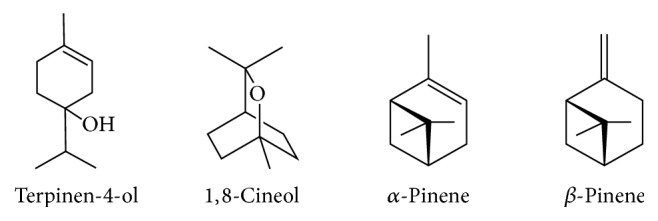
Main substances present in the essential oils from leaves of* A. zerumbet *(terpinen-4-ol; 1,8-cineol) and* A. vittata *(*α*-pinene; *β*-pinene).

Considering the mortality, the groups treated with OLALPVIT and OLALPZER were significantly different in comparison with the controls. In the first 10 min, no insect died in the control groups, but 73.3% of mortality was observed for the group treated with OLALPVIT and 83.3% of mortality for the group treated with OLALPZER, both at 125 *μ*g/*μ*L (Table 4). The insecticidal activity of the major components of these oils was tested against* R. nasutus* fifth-instar nymphs at the same concentration present in the essential oils. Terpinen-4-ol (Figure 3) at 25 *μ*g/*μ*L, corresponding to 20% of OLALPZER, and *β*-pinene (Figure 3) at 44 *μ*g/*μ*L, corresponding to 35% of OLALPVIT, caused 100% of mortality of* R. nasutus* fifth-instar nymphs, showing that these components could be the bioactive targets of the* A. zerumbet *and* A. vittata* essential oils. Quite the contrary, 1,8-cineol and *α*-pinene (Figure 3) did not seem to have effect on the mortality of* R. nasutus* fifth-instar nymphs at the same concentrations; they were present in the essential oils tested.

## 4. Conclusions

The GC-MS analyses of the essential oils from fresh leaves of* A. zerumbet* (OLALPZER) and* A. vittata *(OLALPVIT) tested against* R. nasutus* fifth-instar nymphs demonstrated that the main constituent of OLALPZER was terpinen-4-ol (19.7%), while the main constituent of OLALPVIT was *β*-pinene (35.3%). Both essential oils exhibited antifeedant activity at 125 *μ*g/*μ*L, since the groups treated with these oils did not feed throughout the experiment. However, a significant influence on the development time of* R. nasutus* from fifth-instar nymphs to adult and longevity of adults in the group treated with OLALPZER in comparison to control groups was not observed. Triatomines treated with OLALPVIT died before reaching adult stage. In the fifth 10 minutes of exposition, OLALPVIT and OLALPZER at 125 *μ*g/*μ*L provoked 73.3% and 83.3% of mortality, respectively. The main constituent of OLALPZER, terpinen-4-ol, at 25 *μ*g/*μ*L, and the main constituent of OLAPVIT, *β*-pinene, at 44 *μ*g/*μ*L, caused mortality of 100%, suggesting that they are the main reason for the insecticidal activity of* A. zerumbet* and* A. vittata *essential oils. These results suggest the potential use of these essential oils in* R. nasutus* control and further studies are needed to explore the economic investment in this plant.

## Figures and Tables

**Table 1:** Chemical composition of the essential oils extracted of from fresh leaves of *A. zerumbet* (OLALPZER) and *A. vittata* (OLALPVIT) determined by GC-MS analyzes.

Substances	Retention time	OLALPZER	OLALPVIT
	(min)	Area (%)	Area (%)
*α*-Thujene	12.6	3.7	-
*α*-Pinene	12.9	1.7	10.1
Camphene	13.6	0.2	-
Sabinene	14.7	13.8	1.2
*β*-Pinene	14.8	3.0	35.3
*β*-Myrcene	15.4	1.1	-
Phellandrene	16.1	0.3	-
*α*-Terpinene	16.6	4.0	-
*ρ*-Cymene	16.9	4.8	-
Limonene	17.1	1.8	1.5
1,8-Cineol	17.2	15.3	-
*α*-Terpinolene	-	2.0	-
*cis*-Sabinene	20	2.2	-
Terpinen-4-ol	23.2	19.7	-
*α*-Cubebene	29.0	-	2.1
Copaene	30	-	2.4
*β*-Cubebene	30.4	-	2.4
*β*-Caryophyllene	31.5	3.2	1.0
*β*-Gurjunene	32.4	-	1.7
Aromadendrene	32.8	-	0.7
*cis*-Muurola-4(14),5-diene	33.1	-	0.6
Germacrene-D	33.4	-	0.6
epi-Bicyclosesquiphellandrene	33.8	-	1.0
epi-Cubebol	33.9	-	5.0
Calamenene	34.7	-	2.0
Cadina-1(2),4-diene	35.0		0.8
Viridiflorol	37.2		1.75
1-epi-Cubenol	37.8		1.43
1,10-di-epi-Cubenol	38.2		1.24
Caryophyllene oxide	36.5	1.9	-
Others	-	21.2	27.2

**Table 2:** Mass difference (mg) before and after feeding observed in *R. nasutus* treated with OLALPVIT and OLALPZER at 125 *µ*g/*µ*L under laboratory conditions.

Groups (treatments)	1st week		2nd week		3rd week		4th week		5th week		6th week	
	*χ* ± SD	IV	*χ* ± SD	IV	*χ* ± SD	IV	*χ* ± SD	IV	*χ* ± SD	IV	*χ* ± SD	IV
Pure control	10.6 ± 4.5^a^	0–114	7.8 ± 12.7^a^	0–14	16.0 ± 26.7^a^	0–104	28.9 ± 41.3^a^	0–124	2.6 ± 5.8^a^	0–24	12.0 ± 26.6^a^	0–107
DMSO control	8.8 ± 2.9^a^	0–54	6.4 ± 12.7^a^	0–58	21.7 ± 41.1^a^	0–140	14.5 ± 24.2^a^	0–89	5.7 ± 9.2^a^	0–29	3.6 ± 7.3^a^	0–27
OLALPVIT	0.0 ± 0.0^b^	0-0	0.0 ± 0.0^a^	0-0	0.0 ± 0.0^b^	0-0	0.0 ± 0.0^b^	0-0	0.0 ± 0.0^a^	0-0	0.0 ± 0.0^a^	0-0
OLALPZER	0.0 ± 0.0^b^	0-0	0.0 ± 0.0^a^	0-0	0.0 ± 0.0^b^	0-0	0.0 ± 0.0^b^	0-0	0.0 ± 0.0^a^	0-0	0.0 ± 0.0^a^	0-0

*χ*, means; SD, standard deviation; IV, interval variation (range). Means followed by the same letter within each column are not significantly different according to Tukey's test (*p* < 0.05).

**Table 3:** Development of *R. nasutus* (fifth-instar nymphs to adult) and longevity of adults treated with OLALPVIT and OLALPZER at 125 *µ*g/*µ*L.

Groups	Development (days)		Longevity (days)	
	*χ* ± SD	IV	*χ* ± SD	IV
Without treatment	43.1 ± 17.7^a^	16–79	31.5 ± 10.9^a^	19–56
DMSO	39.9 ± 17.4^a^	7–79	26.1 ± 13.7^a^	12–70
OLALPVIT	ND	ND	ND	ND
OLALPZER	18.5 ± 23.3^a^	2–35	22 ± 21.2^a^	7–37

*χ*, means; SD, standard deviation; ND, not determined; IV, interval variation (range). Means followed by the same letter within each column are not significantly different according to Tukey's test (*p* < 0.05).

**Table 4:** Mortality of *R. nasutus* fifth-instar nymphs treated with OLALPVIT, OLALPZER (125 *µ*g/*µ*L), and their main constituents, terpinen-4-ol (25 *µ*g/*µ*L), 1,8-cineol (19 *µ*g/*µ*L), *α*-pinene (12.5 *µ*g/*µ*L), and *β*-pinene (44 *µ*g/*µ*L).

Groups	Mortality up to 10 min (%)	Mortality between 10 min and 72 h (%)
Without treatment	0	0
DMSO	0	0
OLALPVIT	73.3	6.7
OLALPZER	83.3	3.3
Terpinen-4-ol	100	0
1,8-Cineol	0	0
*α*-Pinene	0	0
*β*-Pinene	100	0
